# Stent-assisted coiling vs. coiling alone of ruptured tiny intracranial aneurysms: A contemporary cohort study in a high-volume center

**DOI:** 10.3389/fneur.2022.1076026

**Published:** 2022-12-06

**Authors:** Guanghao Zhang, Yina Wu, Yanpeng Wei, Gaici Xue, Rundong Chen, Nan Lv, Xiaoxi Zhang, Guoli Duan, Ying Yu, Qiang Li, Yi Xu, Qinghai Huang, Pengfei Yang, Qiao Zuo, Jianmin Liu

**Affiliations:** Neurovascular Center, Changhai Hospital, Naval Medical University, Shanghai, China

**Keywords:** endovascular treatment, tiny ruptured intracranial aneurysms, low-profile visualized intraluminal support stent, procedure-related complications, vascular disorders

## Abstract

**Objective:**

This study aims to compare the safety and efficacy of stent-assisted coiling (SAC) with those of coiling alone (CA) for the treatment of ruptured tiny intracranial aneurysms.

**Methods:**

We enrolled 245 patients with ruptured tiny intracranial aneurysms treated with coil embolization. Patients were grouped into SAC and CA groups. Baseline characteristics, periprocedural complications, clinical outcomes, and angiographic results were compared between the two groups. In addition, a subgroup analysis was conducted in the SAC group, and patients were regrouped into low-profile visualized intraluminal support (LVIS) and laser-cut groups to compare the perioperative procedure-related complications and clinical and angiographic follow-up outcomes.

**Results:**

All baseline characteristics were equivalent between the two groups except for aneurysm size and dome-to-neck aspect ratio. The rates of overall procedure-related complications, intraprocedural rupture, postoperative early rebleeding, intraprocedural thrombosis, postprocedural thrombosis, and procedure-related mortality were comparable between the two groups (*P* = 0.105, 0.145, 0.308, 1.000, 1.000, 0.160, respectively). Nevertheless, the rate of hemorrhagic complication in the SAC group was significantly higher (*P* = 0.023). The angiographic follow-up outcomes showed that the SAC group had a higher complete occlusion rate and lower recurrence rate (88.2 vs. 67.1%, 5.4 vs. 15.2%, *P* = 0.001). The clinical outcomes at discharge and follow-up between the two groups demonstrated no significant differences (*P* = 0.192 and *P* = 0.085, respectively). For subgroup analysis, LVIS stents were associated with a significantly higher rate of complete occlusion (*P* = 0.014) and a lower rate of intraprocedural rupture (*p* = 0.021). Moreover, multivariate analysis showed that there were no predictors for the overall, hemorrhagic, and ischemic procedure-related complications, while Raymond class was an independent predictor of retreatment (OR = 3.508, 95% CI 1.168–11.603; *P* = 0.029).

**Conclusion:**

Stent-assisted coiling may increase the incidence of hemorrhagic events with favorable angiographic results and comparable clinical outcomes compared with stand-alone coiling. Nevertheless, LVIS stent could improve the safety compared with lazer-cut stent. Simultaneously, considering the better long-term effect, LVIS stent-assisted coiling may be a preferable choice for ruptured tiny intracranial aneurysms.

## Introduction

Subarachnoid hemorrhage (SAH) caused by ruptured intracranial aneurysms is one of the most common cerebrovascular diseases (1). Among patients with ruptured aneurysms, 6.2–15.1% are tiny intracranial aneurysms (2, 3). Notably, the unique structural characteristics of tiny intracranial aneurysms, such as very small size, thin aneurysm wall, and relatively wide neck, make it difficult and challenging for both clipping and endovascular treatment (4).

With the advances in neuroimaging and endovascular devices, several recent studies corroborated comparable effectiveness and better prognosis when using endovascular treatment as compared to microsurgical clipping (5, 6) for ruptured tiny intracranial aneurysms. Simultaneously, previous studies indicated that the stent-assisted coiling (SAC) technique was associated with a higher complete occlusion rate and lower recurrence rate at follow-up compared with coiling alone (CA) in ruptured intracranial aneurysms (7, 8). However, studies on aneurysm occlusion, recurrence, and procedural complication rates of SAC treatment for ruptured tiny intracranial aneurysms were limited and heterogeneous (9, 10). The safety and efficacy of SAC in the treatment of ruptured tiny intracranial aneurysms need to be further investigated.

Since its debut as an endovascular aid, Neuroform stents (Stryker, Kalamazoo, MI, USA) were quickly followed by other stents, and each stent targets aneurysms of specific shapes and parent patterns (11). Given the diversity of stents available, tailored therapeutics may be employed based on the angioanatomic conditions and configurations to improve perioperative safety and long-term sustainability. The low-profile visualized intraluminal support (LVIS) device (MicroVention, Tustin, CA, USA) is a self-expandable braided stent with higher metal coverage and less porosity than laser-cut stents (Enterprise, Neuroform stents, Solitaire stent, etc.). Our previous efforts suggested that perioperative procedure-related complications and aneurysm occlusion rates in intracranial aneurysms proved more favorable when using LVIS stents (7, 12). However, whether similar complications, angiographic outcomes, and clinical outcomes were achieved in ruptured tiny intracranial aneurysms subjected to LVIS SAC is not well-known.

In the present study, we compared SAC with CA in a high-volume center to further evaluate the safety and efficacy of SAC for the treatment of acutely ruptured tiny intracranial aneurysms. Then, we focused on the safety and efficacy of different stents, making a direct comparison between LVIS and laser-cut stents to comment on the periprocedural complications and occlusive status at follow-up. We further analyzed the in?uential factors associated with the perioperative complications and recurrence rate of these patients.

## Methods

### Study design

In this retrospective study, we collected the clinical data of 245 consecutive patients who were hospitalized for ruptured tiny intracranial aneurysms and treated endovascularly between January 2014 and December 2018 in our center. Among them, 93 patients underwent SAC, and 152 patients underwent CA. The study protocol was approved by the Ethics Committee of the Shanghai Changhai Hospital. Written informed consent was waived given the retrospective nature of the analysis.

The inclusion criteria were as follows: (1) Aneurysm rupture diagnosed by CT or lumbar puncture and ruptured tiny intracranial aneurysms diagnosed *via* digital subtraction angiography (DSA); (2) aneurysm treated within 28 days after SAH; (3) maximum aneurysm diameter was ≤ 3 mm *via* performing 3D rotational angiography; and (4) aneurysm treated by SAC or CA (including balloon-assisted coiling). The exclusion criteria were as follows: (1) Fusiform, traumatic, dissecting, pseudo-, and blood blister-like aneurysms; (2) reruptured aneurysms with previous treatment; (3) parent artery occlusion, simple stent placement alone, and coiling with other embolization materials; (4) multiple aneurysms but failed to identify the ruptured one; (5) staged stent placement; and (6) incomplete clinical data.

We collected baseline data from the patients, including age, sex, medical history, aneurysm location, preoperative Hunt-Hess grade, aneurysm size, Modified Fisher Grading Scale, and Modified Rankin Scale (mRS) score. In addition, other clinical data were also obtained on aneurysm size, dome-to-neck aspect ratio, and location.

### An endovascular procedure and medications

All included patients were treated by eight endovascular neurosurgeons with experience of more than 10 years. All procedures were performed under general anesthesia. After systemic heparinization, rotational DSA and 3D reconstruction were performed routinely. The size of the aneurysm and the diameter of the distal and proximal aneurysmal parent artery were measured to select the appropriate coil and stent. During the procedures, the activated clotting time was maintained at 2–3 times the baseline level. All stents (LVIS, MicroVention Terumo, USA; Enterprise, Cordis, USA; Solitaire, Covidien, USA; Neuroform, Boston Scientific, USA) and coils were deployed according to the standard procedure recommended by the manufacturer. After the decision to deploy a stent was made, a loading dose of aspirin (300 mg) and clopidogrel (300 mg) was given orally or rectally to patients who had stent-assisted coil embolization. A loading dose (5 μg/kg for 3 min) of glycoprotein IIb/IIIa inhibitor (tirofiban; Grand Pharma, China) was intravenously injected to prevent platelet aggregation before stent release and maintained at a rate of 0.075 μg/kg/min for 6 h. For patients who underwent SAC, dual-antiplatelet therapy (100 mg aspirin and 75 mg clopidogrel) was maintained for 6 weeks after the procedure, followed by aspirin (100 mg) alone for at least 12 months. The antiplatelet protocol was adjusted according to the angiographic results and the patient's results of thromboelastography during the follow-up period. In case of acute thrombosis in the stent during the procedure, tirofiban was injected intraarterially at a dose of 0.075 ug/kg/min through a microcatheter. If intraprocedural rupture occurred, heparin was neutralized by using protamine sulfate immediately, and dense embolization of the aneurysm was performed as much as possible through packing coils quickly.

### Clinical and angiographic follow-up

Clinical follow-up was typically scheduled at the 3rd, 6th, and 12th months, and the results were evaluated using the modified Rankin Scale (mRS). Favorable clinical outcomes were defined as an mRS score of 0 to 2, and poor clinical outcomes were defined as an mRS score of 3 to 6. Angiographic follow-up was assessed by magnetic resonance angiography or DSA routinely in the 6th month after the procedure and yearly thereafter and was classified using the Raymond–Roy occlusion classification. The cases in the CA group who underwent salvage stent placement because of coil protrusion were counted as the SAC group at follow-up.

### Statistical analysis

Statistical analysis was performed using R version 4.1.3 software. Independent samples *t*-test, Pearson's χ^2^ test, Fisher's exact test, or non-parametric test was used for statistical analysis as appropriate. Categorical variables were presented as frequency, and continuous variables were expressed as mean ± standard deviation (x ± s) for normally distributed variables and median (IQR) for non-normally distributed variables, respectively. Univariate and multivariate analyses were performed to identify the association between procedure-related complications and predictive risk factors. The univariate analysis cutoff for inclusion in the multivariate analysis was *p* < 0.20. A *p*-value of < 0.05 was considered statistically significant.

## Results

### Baseline characteristics

A total of 245 patients with ruptured tiny intracranial aneurysms were enrolled in this study. The SAC group and CA group were statistically comparable with respect to age, sex, disease history, location, neck size, parent artery configuration, WNFS, Hunt-Hess, modified Fisher grading, interval between aneurysm rupture and procedure and surgery (Table 1). The SAC group had a smaller aneurysm size [median (IQR) 2.3 (1.9–2.6) vs. 2.5 (2.2, 2.8)] and a bigger dome-to-neck aspect ratio [1.180 (1.0–1.4) vs. 1.4 (1.1–1.7)] (Table 1).

**Table 1:** Characteristics of patients at baseline.

**Characteristics**	**Group**		***P-*value**
	**SAC** ** (*n* = 93)**	**CA** ** (*n* = 152)**	
Age, yrs	55.366 (10.410)	55.855 (12.592)	0.753
**Sex**			
Female	59 (63.44)	94 (61.84)	0.909
Male	34 (36.56)	58 (38.16)	
Hypertension, *n* (%)	44 (47.31)	82 (53.95)	0.381
Coronary heart disease, *n* (%)	3 (3.23)	5 (3.29)	1.000
Diabetes mellitus, *n* (%)	7 (7.53)	10 (6.58)	0.981
Smoking (%)	10 (10.75)	20 (13.16)	0.721
intracranial hematoma (%)	10 (10.75)	17 (11.18)	
Size(median [IQR])	2.300 [1.940, 2.620]	2.500 [2.152, 2.762]	0.007
Neck (median [IQR])	1.900 [1.500, 2.260]	1.815 [1.400, 2.092]	0.160
Dome-to-neck aspect ratio (median [IQR])	1.180 [1.010, 1.360]	1.360 [1.128, 1.662]	0.000
Intraventricular hematoma (%)	29 (31.18)	48 (31.58)	
**Location (%)**			
ICA	20 (21.51)	14 (9.21)	0.074
PcomA	20 (21.51)	26 (17.11)	
ACA	6 (6.45)	14 (9.21)	
AcomA	32 (34.41)	71 (46.71)	
MCA	9 (9.68)	19 (12.50)	
PC	6 (6.45)	8 (5.26)	
**Parent artery configuration**			
Bifurcation	47 (50.54)	87 (57.24)	0.374
Side wall	46 (49.46)	65 (42.76)	
**WFNS (%)**			
1	65 (69.89)	106 (69.74)	0.306
2	14 (15.05)	13 (8.55)	
3	3 (3.23)	3 (1.97)	
4	8 (8.60)	20 (13.16)	
5	3 (3.23)	10 (6.58)	
**Hunt-Hess (%)**			
1	11 (11.83)	18 (11.84)	0.613
2	47 (50.54)	68 (44.74)	
3	29 (31.18)	49 (32.24)	
4	6 (6.45)	17 (11.18)	
**modified Fisher grade (%)**			
1	18 (19.35)	35 (23.03)	0.221
2	62 (66.67)	82 (53.95)	
3	9 (9.68)	26 (17.11)	
4	4 (4.30)	9 (5.92)	
**Interval between aneurysm rupture and procedure**			
<72 h	60 (64.52)	111 (73.03)	0.303
72 h−14 d	30 (32.26)	35 (23.03)	
>14 d	3 (3.23)	6 (3.95)	
**Surgery**			
EVD	9 (9.68)	12 (7.89)	1.000
VP shunt	3(3.23)	4(2.61)	1.000
Other	3(3.23)	2(1.32)	0.373

mm, millimeter; ICA, internal carotid artery; ACA, anterior cerebral artery; MCA, middle cerebral artery; ACoA, anterior communicating artery; PCoA, posterior communicating artery; PC, posterior circulation; EVD, external ventricular drainage; VP shunt, ventriculoperitoneal shunt.

Unless indicated otherwise, data are presented as the number of patients (%).

### Immediate embolization results and clinical outcomes at discharge

All stents were successfully deployed in the SAC group, whereas the salvage stent technique was used in 1 case (1.0%, 1/93) in the CA group due to the coil protrusion. The immediate angiographic results showed that Raymond class I was achieved in 59 cases (63.4%, 59/93), Raymond class II–III in 9 cases (9.7%, 9/93), and Raymond class III in 25 cases (26.9%, 25/93) in the SAC group, compared with 85 cases (55.9%, 85/152), 41 cases (27.0%, 41/152), and 26 cases (17.1%, 26/152) in the CA group, respectively, which showed no statistically significant difference between the two groups (*P* = 0.078). A total of 89.25% (83/93) of patients in the SAC group and 82.2% (125/152) of patients in the CA group had favorable neurological outcomes at discharge, showing no statistically significant difference between the two groups (*P* = 0.193) (Table 2).

**Table 2:** Angiographic and clinical outcomes for patients treated with SAC and CA.

**Outcomes**	**Group**		***P*-value**
	**SAC**	**CA**	
	**(*n* = 93)**	**(*n* = 152)**	
**Immediate embolization result**			
Raymond class I	59 (63.44)	85 (55.92)	0.246
Raymond class II-III	34 (36.56)	67 (44.08)	
**Clinical outcome at discharge**			
mRS score 0 to 2	83 (89.25)	125 (82.24)	0.193
mRS score 3 to 6	10 (10.75)	27 (17.76)	
**Angiographic follow-up**			
Complete occlusion	66 (89.19)	57 (67.06)	0.001
Improvement	2 (2.70)	1 (1.18)	
Stability	2 (2.70)	14 (16.47)	
Recurrence	4 (5.4)	13 (15.29)	
Retreatment	1 (1.35)	9 (10.59)	0.094
**Clinical follow-up** ^**a**^			
mRS score 0 to 2	80 (97.56)	124 (90.51)	0.085
mRS score 3 to 6	2 (2.44)	13 (9.49)	
**Clinical follow-up** ^**b**^			
mRS score 0 to 2	80 (88.89)	124 (84.35)	0.432
mRS score 3 to 6	10 (11.11)	23 (15.65)	

a Excluding patients who died at discharge.

b Including patients who died at discharge.

In the SAC group, Raymond class I and Raymond class II-III were achieved in 45 (67.2%) and 22 (22.84%) patients treated with LVIS and were achieved in 14 (53.9%) and 12 (46.2%) patients treated with laser-cut stents, which showed no significant difference between the two groups (*P* = 0.33); 59 (88.1%) patients treated with LVIS had an mRS of 0 to 2 compared with 24 (92.3%) patients treated with laser-cut stents without statistical significant difference (*P* = 0.83) (Table 3).

**Table 3:** Angiographic and Clinical Outcomes for patients treated with LVIS and laser-cut stent.

**Outcomes**	**Group**		***P*-value**
	**LVIS** ** (*n* = 67)**	**Laser-cut** ** (*n* = 26)**	
**Immediate embolization result**			
Raymond class I	45 (67.16)	14 (53.85)	0.339
Raymond class II-III	22 (32.84)	12 (46.15)	
**Clinical outcome at discharge**			
mRS score 0 to 2	59 (88.06)	24 (92.31)	0.825
mRS score 3 to 6	8 (11.94)	2 (7.69)	
**Angiographic follow-up**			
Complete occlusion	51 (94.44)	15 (75.00)	0.014
Improvement	0 (0.00)	2 (10.00)	
Stability	2 (3.70)	0 (0.00)	
Recurrence	2 (3.6)	2 (10.0)	
Retreatment	0 (0.00)	1	0.257
Clinical follow-up^a^			
mRS score 0 to 2	58 (96.7)	22 (100)	1.000
mRS score 3 to 6	2 (3.33)	0 (0)	
Clinical follow-up^b^			
mRS score 0 to 2	58 (87.9)	22 (91.7)	1.000
mRS score 3 to 6	8 (12.1)	2 (8.3)	

### Periprocedural complications and mortality

Overall, perioperative procedure-related complications occurred in 11 patients (11.8%, 11 of 93) in the SAC group and in eight patients (5.3%, 8 of 152) in the CA group, which were comparable (*p* = 0.106). Specifically, the hemorrhagic complication rate of the SAC group was higher than those of the CA group (*P* = 0.023), while the ischemic complications were comparable (*P* > 0.99).

For hemorrhagic complications, intraprocedural rupture, aneurysm rebleeding, and surgical procedure-related hemorrhagic events occurred in five patients (3.0%, 4 of 133), three patients (1.5%, 2 of 133), and no patient (0.8%, 1 of 133) of the SAC group and two patients (1.0%, 3 of 289), one patient (1.4%, 4 of 289), and no patient of the CA group, respectively (*P* = 0.145, *P* = 0.308, and *P* > 0.99, respectively).

For ischemic complications, intraprocedural thrombosis and postprocedural thrombosis occurred in two patients (2.2%, 2/93) and one patient (1.1%, 1/93) of the SAC group, respectively, compared with four patients (2.6%, 4/152) and no patient of the CA group, respectively (*P* > 0.99 and *P* = 0.804, respectively).

Procedure-related mortality rates for patients who had the above complications were 5.4% (5/92) in the SAC group (four cases of aneurysm rebleeding and one case of postprocedural thrombosis) and 1.97% (3/152) in the CA group (two cases of intraoperative rupture and one case of postoperative rebleeding). No coil protrusion into the parent artery occurred (Table 4).

**Table 4:** Perioperative Complications for patients treated with SAC and CA.

**Perioperative complications**	**Group**		***P*-value**
	**SAC** ** (*n* = 93)**	**CA** ** (*n* = 152)**	
Procedure-related complications	11 (11.8)	8 (5.3)	0.105
**Hemorrhagic**	8 (8.60)	3 (1.97)	0.023
Intraprocedural rupture	5 (5.38)	2 (1.32)	0.145
Postprocedural early rebleeding	3 (3.23)	1 (0.66)	0.308
Surgical procedure-related hemorrhagic event	0	0	1
**Ischemic**	3 (3.23)	5 (3.29)	1
Intraprocedural thrombosis	2 (2.15)	4 (2.63)	1
Postprocedural thrombosis	1 (1.1)	1 (0.7)	1
Coil protrusion	0	0	1
Salvage technique	0	1 (0.7)	1
Cerebral vasospasm	6 (6.5)	14 (9.2)	0.599
Procedure-related mortality	5 (5.38)	3 (1.97)	0.160

Unless indicated otherwise, data are presented as the number of patients (%).

Among the patients who were treated with SAC, overall procedure-related complications were more common in patients with laser-cut stents than in those with LVIS without statistical significance (23.1%, 6/26 vs. 7.5%, 5/67, *P* = 0.067). The hemorrhagic complication rates in the LVIS group (4.4%, 5/67) were significantly lower compared with the laser-cut group (23.1%, 6/26) (*P* = 0.031), while the ischemic complication rates were similar. Regarding hemorrhagic complications, an intraprocedural rupture occurred in one patient in the LVIS group and four patients in the laser-cut group (*P* = 0.021). Postprocedural early rebleeding occurred in 2 patients (1.5%) in the LVIS group and one patient (3.8%) in the laser-cut group, but the difference was not statistically significant (*P* > 0.99). For ischemic complications, intraprocedural thrombosis and postprocedural thrombosis occurred in two patients (3.0%) and no patient in the LVIS group, compared with no patient and one patient (3.8%) in the laser-cut group (*P* > 0.99 and =0.280, respectively). Patients with LVIS carried a slightly lower procedure-related mortality (3.0 vs. 11.5%); however, this difference was not statistically significant (*P* = 0.131) (Table 5).

**Table 5:** Perioperative Complications for patients treated with LVIS and laser-cut stent.

**Perioperative complications**	**Group**		***P*-value**
	**LVIS** ** (*n* = 67)**	**Lazer-cut** ** (*n* = 26)**	
Procedure-related complications	5 (7.5)	6 (23.1)	0.067
**Hemorrhagic**	3 (4.4)	5 (19.2)	0.036
Intraprocedural rupture	1 (1.5)	4 (15.4)	0.021
Post-procedural early rebleeding	2 (3.0)	1 (3.8)	1.000
Surgical procedure-related hemorrhagic event	0	0	1.000
**Ischemic**	2 (3.0)	1 (3.8)	1.000
Intraprocedural thrombosis	2 (3.0)	0	1.000
Postprocedural thrombosis	0	1 (3.8)	0.280
Cerebral vasospasm	4 (6.0)	2 (7.7)	0.671
Procedure-related mortality	2 (3.0)	3 (11.5)	0.131

### Clinical and angiographic follow-up

**Figure 1:**
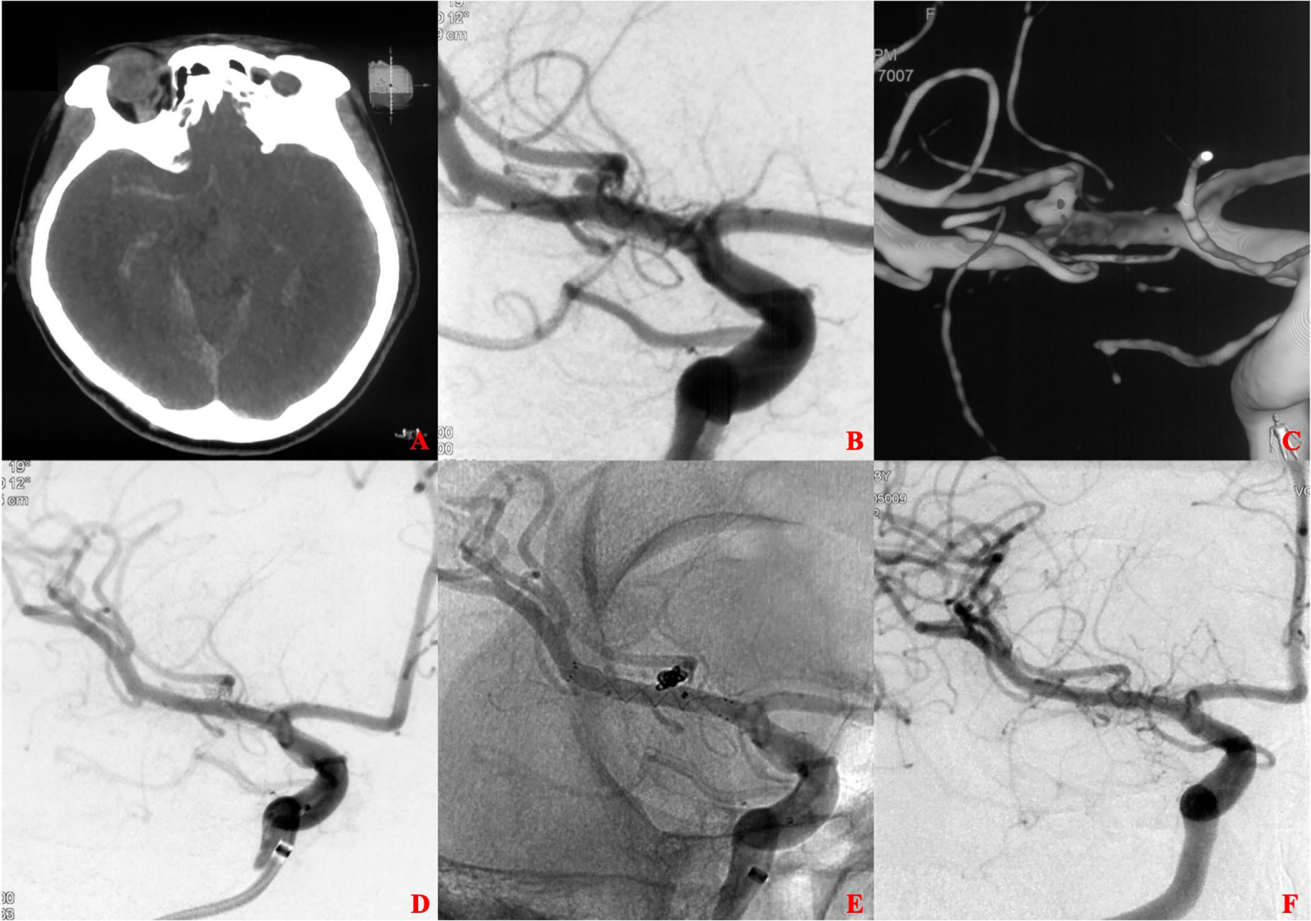
A ruptured tiny middle cerebral artery (MCA) intracranial aneurysm treated with stent-assisted coiling (SAC). **(A)** The patient was admitted with spontaneous subarachnoid hemorrhage. **(B,C)** Cerebral angiography and 3D reconstruction revealed a tiny MCA bifurcation aneurysm. **(D,E)** The aneurysm was treated with SAC embolization using an LVIS stent (3.5 × 15 mm). Immediate angiography showed that the aneurysm was completely occluded. **(F)** 13 months later, angiographic images showed complete occlusion of the aneurysm without in-stent artery stenosis.

**Figure 2:**
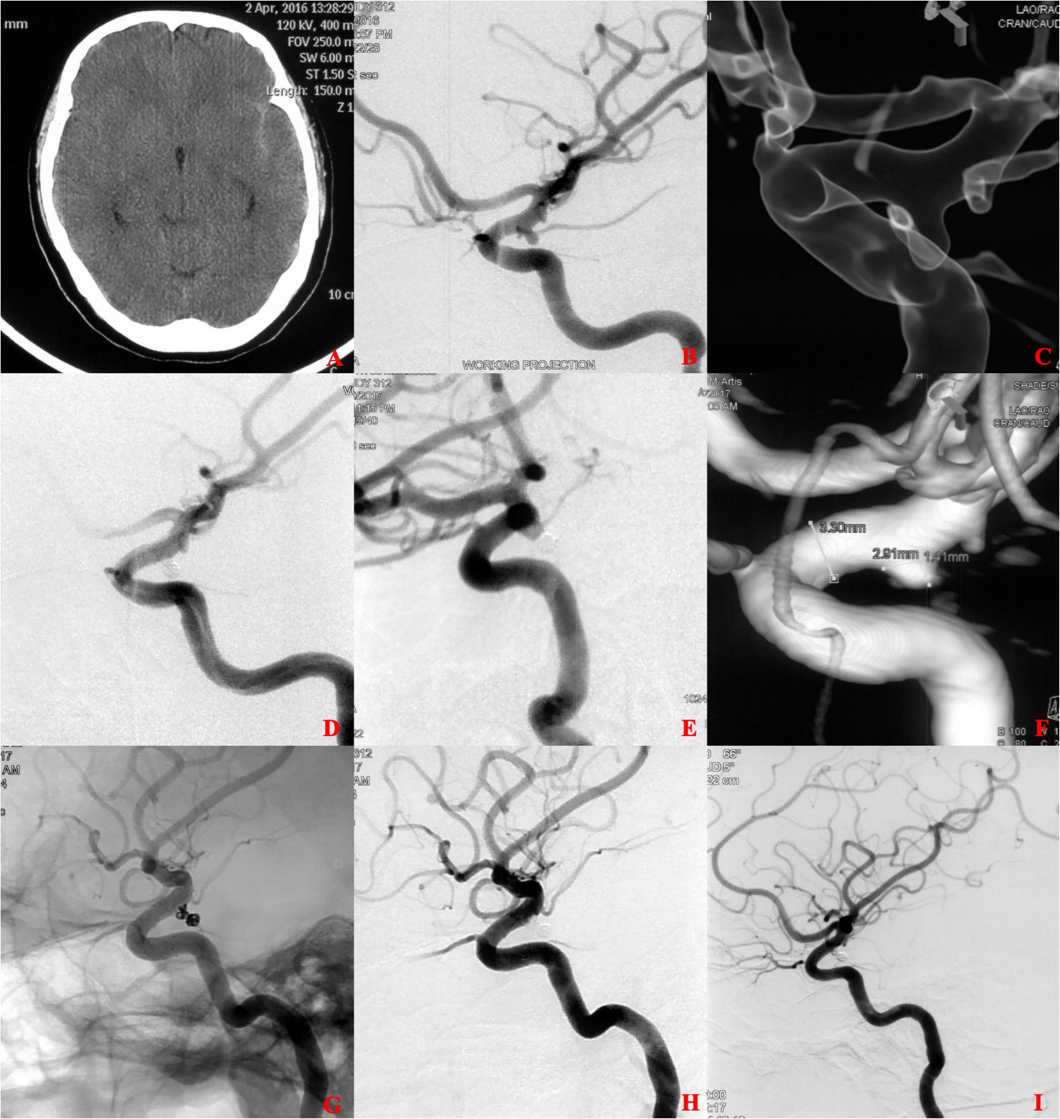
A ruptured tiny posterior communicating artery (PCOM) aneurysm treated with coiling alone (CA). **(A)** The patient was admitted with spontaneous subarachnoid hemorrhage. **(B,C)** Cerebral angiography and 3D reconstruction revealed a tiny PCOM aneurysm. **(D)** The aneurysm was treated with coiling embolization only. Immediate angiography showed that the aneurysm was completely occluded. **(E,F)** 6 months later, angiographic images showed postoperative recurrence of the aneurysm. **(G,H)** The aneurysm retreated with additional coiling embolization and an LVIS stent (4.5 × 15 mm). Immediate angiography showed that the aneurysm was completely occluded. **(I)** 12 months later, angiographic images showed complete occlusion of the aneurysm.

In total, eight patients in the SAC group and 10 patients in the CA group passed away before discharge. Therefore, a total of 227 patients survived the initial SAH at discharge. Among them, 159 patients (70.0%, 159 of 227) had been followed up angiographically, ranging from 6 to 2,260 days (mean, 423 days). Angiographic follow-up data demonstrated that complete occlusion, improvement, stability, recurrence, and retreatment were achieved in 66 cases (89.2%, 66 of 74), two cases (2.7%, 2 of 74), two cases (2.7%, 2 of 74), four cases (5.4%, 4 of 74), and one case (1.4%, 1 of 74), respectively, in the SAC group compared with 57 cases (67.1%, 57/85), one case (1.2%, 1/85), 14 cases (16.5%, 14/85), 13 cases (15.3%, 13/85), and nine cases (10.6%, 9/85), respectively, in the CA group. The SAC group showed a higher complete occlusion rate and a lower recurrence rate than the CA group (*P* < 0.001) (Table 2, Figures 1, 2).

Among these surviving patients, 219 patients (96.5%, 219 of 227) had been followed up clinically for 180 to 2,304 days (mean, 1,305 days); of which, four patients (4.7%, 4 of 86) had poor neurological outcomes (mRS score of 3–6) in the SAC group, whereas 13 patients (10.0%, 13 of 130) had poor neurological outcomes in the CA group (*P* = 0.242). All parent arteries were patent without clinically significant in-stent stenosis, and no aneurysm rebleeding or thrombosis events occurred during the follow-up period (Table 2).

For the SAC group, at least one angiographic follow-up was performed in 54 patients (80.6%, 54/67) in the LVIS group and 21 patients (76.9%, 20/26) in the laser-cut group. Follow-up angiograms showed complete occlusion in 51 cases (94.44%,51/54), improvement in no (0%, 0/54) case, stability in 2 (3.7%, 2/54) cases, and recurrence in one case (1.85%, 1/54) in the LVIS group, compared with 15 cases (75.0%, 15/20), two cases (10.0%, 2/20), no case (0%, 0/20), 3 cases (15.0%, 3/20) in the laser-cut group, showing statistically significant difference between the two groups (*P* = 0.014) (Table 3).

### Multivariate analysis for risk factors of procedure-related complications

Univariate analysis showed that the intracranial hematoma (*P* = 0.018), intraventricular hematoma (*P* = 0.157), sidewall (*P* = 0.18), and SAC group (*P* = 0.074) were associated with overall perioperative procedure-related complications; intracranial hematoma (*P* = 0.001), intraventricular hematoma (*P* = 0.028), external ventricular drainage (*P* = 0.11), and SAC group (*P* = 0.074) were associated with hemorrhagic procedure-related complications; smoking (*P* = 0.105), size (*P* = 0.102), SAC group (*P* = 0.054), Raymond class (*P* = 0.027), and the interval between aneurysm rupture and procedure (*P* = 0.172) were associated with retreatment; and no risk factor was associated with ischemic procedure-related complications. Moreover, multivariate analysis showed that there were no predictors for the overall, hemorrhagic, and ischemic procedure-related complications, while Raymond class was an independent predictor of retreatment (OR = 3.508, 95% CI 1.168–11.603; *P* = 0.029).

## Discussion

In this single-center retrospective cohort study, perioperative complications and treatment outcomes of tiny ruptured intracranial aneurysms were compared between the SAC group and the CA group. Moreover, in the SAC group, a direct comparison between LVIS and laser-cut stents was conducted to assess the effect between these two stents on the periprocedural safety and occlusive status during follow-up. The procedure-related hemorrhagic complication rate was higher in the SAC group than that in the CA group, whereas the ischemic complication rate was comparable. Moreover, the SAC group showed a significantly higher complete occlusion rate and a significantly lower retreatment rate compared with the CA group at follow-up. The favorable clinical outcome rate was similar in both groups. Further analysis indicated that although the univariate analysis showed an increased incidence of procedure-related hemorrhagic events in the SAC group, the multivariate analysis showed that SAC was not an independent risk factor. Besides, the multivariate analysis also showed that SAC was not a predictor for overall perioperative procedure-related complications and ischemic procedure-related complications of acutely ruptured tiny intracranial aneurysms. Among the SAC group, we observed significantly lower overall procedure-related complications, hemorrhagic complications, and intraprocedural aneurysm rupture in the LVIS group than those in the laser-cut group. In addition, follow-up angiographic results suggested that LVIS SAC was associated with a higher occlusion rate compared with laser-cut SAC. Favorable clinical outcomes at discharge and during long-term follow-up were comparable between the two groups of different stents. Summarizing these results, SAC might increase the risk of intracranial hemorrhagic events; however, these were mostly minor incidents associated with low morbidity. In addition, the SAC strategy has better long-term angiographic outcomes when compared to the CA strategy. When considering only patients treated with SAC, our cohort showed that LVIS SAC performed more safely and effectively than the laser-cut SAC for the treatment of tiny ruptured intracranial aneurysms.

Consistent with previous reports (9, 13), the majority of tiny aneurysms in our series were wide-necked. To avoid coil protrusion into the parent vessel and subtotal occlusion of the aneurysm, several studies reported that the aneurysm with a wider neck is more likely to use the SAC technique (14, 15). In addition, the very small size of tiny aneurysms limits the operation space of the microcatheter tip and has higher requirements for the stability of delivery systems (16, 17). Therefore, for the aneurysm with a relatively smaller size, to reduce the risk of intraoperative rupture, our center prefers to use SAC. The comparison of background characteristics between SAC and CA groups in our cohort demonstrated the expected differences. The variability of treatment strategy reflects the skill and experience of the operator and highlights the lack of specific evidence on which structural characteristics of RIA are suitable for SAC.

Endovascular treatment-related hemorrhagic events and thromboembolism are the most common complications of morbidity and mortality caused by intravascular treatment of intracranial aneurysms. Ruptured intracranial aneurysms seem to be more susceptible to endovascular treatment-related hemorrhagic events than unruptured lesions (18). In addition, SAC, which requires antiplatelet medication in the setting of acutely ruptured aneurysms, increases the theoretical risk of hemorrhagic complications. A multicenter retrospective cohort confirmed this concern. The authors reported that the aneurysm rebleeding rate in the SAC group was significantly higher than that of the CA group (17.4 vs. 1.9%, *P* < 0.007) (19). A meta-analysis of eight retrospective cohort studies with 909 RIA patients who underwent CA and 499 RIA patients who underwent SAC suggested the incidence of hemorrhagic events increased in the SAC group (OR 1.6, 95% CI 1.1–2.4, *P* = 0.319), but the favorable clinical outcome rate was comparable between the two groups (OR 0.95, 95% CI 0.88–1.02, *P* = 0.338) (20). In the present study, the risk of hemorrhagic complications was significantly higher in the SAC group than in those under CA therapy. In the SAC group, hemorrhagic events occurred in eight patients. Among them, one patient died before discharging due to a poor clinical grade at presentation and comorbidity. Of the remaining seven patients, five had good outcomes (MRS 0–2) at discharge, and two had poor outcomes (mRS 3–6). MRS of all patients did not improve during the follow-up period. In the CA group, three patients experienced hemorrhagic complications and had poor outcomes at discharge. Among them, two patients died due to multiple organ failures during the follow-up period, and one patient had no change in clinical outcome. A total of 86 patients in the SAC group and 122 patients in the CA group received clinical follow-up. The favorable clinical outcome rate at follow-up was similar between the two groups (84/86 vs. 119/122, 97.67 vs. 97.54%, *P* = 1.000), which was consistent with previous studies (7). Another two studies suggested that antiplatelet medication during SAH increased the risk of ventriculostomy-related hemorrhagic complications, but without further impact on the course and outcome of SAH (21, 22). Nevertheless, in our study, probably due to the limited sample size, there were no surgery-related hemorrhagic complications in the two groups, and this issue needs to be further investigated. On the contrary, thromboembolic complications of SAC are also a matter of concern. Several early studies showed that perioperative thromboembolic risk increased in the SAC group (23, 24). However, the recent reports for endovascular treatment of tiny ruptured intracranial aneurysms showed a low thromboembolic complication rate in both SAC and CA groups without a significant difference between them (10, 13). In the present study, our results further confirm this observation.

The performance of each stent type depends on structures or manufacturing processes, showing different behaviors in delivery method, neck protection, and flow diversion. When it comes to the SAC group, various clinical and angiographic outcomes in braided and laser-cut SAC for intracranial aneurysms have been observed in several studies (12, 25, 26). Nevertheless, the performance of these two stent types in terms of perioperative procedure-related complications is still controversial. Ge et al. reported 96 intracranial aneurysms in the braided stent (LVIS) group and 159 aneurysms in the laser-cut stent (Enterprise) group and found that the rate of hemorrhagic complication and thromboembolic events was comparable between the two groups (25). In addition, similar results have been reported in other studies (27, 28). According to our present study, intraprocedural rupture rates of patients treated with SAC proved significantly lower when using LVIS stent (vs laser-cut stent) (*P* = 0.02), and thromboembolism rates were slightly lower without statistical significance (*P* = 1.000). Our previous study on ruptured aneurysms observed similar results regarding periprocedural safety for treated aneurysms involving LVIS and laser-cut stents (12). Compared with laser-cut stents, smaller coils are available to be combined with LVIS stents with smaller mesh to improve the safety of the procedure. This factor may account for the highly statistically significant increase in the rate of intraprocedural rupture for laser-cut stents. To prevent thromboembolism, the modified antiplatelet regimen described in previous studies was adopted in our center (12). In the present study, the thromboembolism rates were comparable between LVIS and laser-cut groups and lower than those reported previously (12, 29, 30).

Our results agree with previous studies showing an immediate complete occlusion rate of 40.6–69.0% and a follow-up complete occlusion rate of 60.0–91.7% after SAC of ruptured tiny aneurysms (13, 31–33). In addition, we observed that the immediate complete occlusion rate in the SAC groups was higher than that in the CA group (63.44% vs. 55.92), although the difference was not significant (*P* = 0.246); the follow-up complete occlusion rate was significantly higher (88.19 vs. 67.06%, *P* = 0.001); and the retreatment rate was significantly lower (1.35 vs. 10.59%, *P* = 0.004) when using SAC treatment. These results are possible due to a continuous thrombosis process toward a more complete occlusion in the SAC group.

The low-profile visualized intraluminal support improves flow diversion effect and promotes reendothelialization due to its higher metal coverage (23%) and smaller mesh (1 mm) compared with laser-cut stents, theoretically, which could promote delayed aneurysm thrombosis and obtain a favorable occlusion rate in long-term follow-up (34). Nevertheless, a recent systematic review showed that the follow-up complete occlusion and recurrence in the LVIS group were comparable with the laser-cut group (*P* = 0.454, 0.056, respectively) (26). Lim et al. (35) reported a cohort study and demonstrated similar outcomes in follow-up and recurrence rates between the LVIS group and the Enterprise group. Our cohort study showed that for ruptured tiny intracranial aneurysms, patients treated with LVIS yielded significantly higher follow-up complete occlusion and lower recurrence rates (*P* = 0.014), while the retreatment rate was lower without statistical significance (*P* = 0.27). The results were similar to those reported by previous studies (25, 28). Notably, although the recurrence rate was higher in the laser-cut group compared with the LVIS group in the present study (10.0%), it was still comparable with previous studies on aneurysms treated with laser-cut stents (25, 36, 37).

The present study has some limitations. First, this study from one single center is non-randomized and retrospective, with an inherent selection bias. Second, our findings need to be interpreted with caution due to the relatively small sample size of each stent group and the low incidence of retreatment.

## Conclusion

Stent-assisted coiling may increase the incidence of hemorrhagic events with favorable angiographic results and similar clinical outcomes compared with stand-alone coiling. Nevertheless, LVIS stent appears to improve the safety compared with lazer-cut stent. Simultaneously, considering the better long-term effect, LVIS SAC may be a preferable choice for ruptured tiny intracranial aneurysms. Prospective studies with larger sample sizes are needed to further confirm the safety and efficacy of the SAC treatment.

## Data availability statement

The original contributions presented in the study are included in the article/supplementary material, further inquiries can be directed to the corresponding authors.

## Ethics statement

The studies involving human participants were reviewed and approved by the Institutional Ethics Committee of the Changhai Hospital of Naval Medical University (No. CHEC2022-149). Written informed consent for participation was not required for this study in accordance with the national legislation and the institutional requirements. Written informed consent was obtained from the individual(s) for the publication of any potentially identifiable images or data included in this article.

## Author contributions

Conception or design of the work: JL and QZ. Acquisition of data: RC, GX, and NL. Analysis of data: QL, XZ, and GD. Interpretation of data: PY and QH. Drafting the work: GZ and YWu. Revising the work: YX, YY, and QZ. Final approval of the version: JL. All authors contributed to the article and approved the submitted version.

## Funding

234 Peak Climbing Program of Changhai Hospital (2020YXK060) Shanghai Shenkang 3-year Action Plan Major Clinical Research Project (SHDC2020CR4037).

## Conflict of interest

The authors declare that the research was conducted in the absence of any commercial or financial relationships that could be construed as a potential conflict of interest.

## Publisher's note

All claims expressed in this article are solely those of the authors and do not necessarily represent those of their affiliated organizations, or those of the publisher, the editors and the reviewers. Any product that may be evaluated in this article, or claim that may be made by its manufacturer, is not guaranteed or endorsed by the publisher.

## References

[1] Macdonald RL Schweizer TA. Spontaneous subarachnoid hemorrhage. Lancet. (2017) 389:655–66. 10.1016/S0140-6736(16)30668-7 27637674

[2] van Rooij WJ Keeren GJ Peluso JPP Sluzewski M. Clinical and Angiographic Results of Coiling of 196 Very Small ( ≤ 3 mm) Intracranial Aneurysms. AJNR Am J Neuroradiol. (2009) 30:835–9. 10.3174/ajnr.A1429 19131407 PMC7051764

[3] Weir B Disney L Karrison T. Sizes of ruptured and unruptured aneurysms in relation to their sites and the ages of patients. J Neurosurg. (2002) 96:64–70. 10.3171/jns.2002.96.1.0064 11794606

[4] Backes D Rinkel GJE Laban KG Algra A Vergouwen MDI. Patient- and aneurysm-specific risk factors for intracranial aneurysm growth: a systematic review and meta-analysis. Stroke. (2016) 47:951–7. 10.1161/STROKEAHA.115.012162 26906920

[5] Zhao B Xing H Fan L Tan X Zhong M Pan Y. Endovascular coiling versus surgical clipping of very small ruptured anterior communicating artery aneurysms. World Neurosurg. (2019) 126:e1246–50. 10.1016/j.wneu.2019.03.074 30898747

[6] Li J Su L Ma J Kang P Ma L Ma L. Endovascular coiling vs. microsurgical clipping for patients with ruptured very small intracranial aneurysms: management strategies and clinical outcomes of 162 cases. World Neurosurg. (2017) 99:763–9. 10.1016/j.wneu.2015.11.079 26732968

[7] Xue G Zuo Q Tang H Zhang X Duan G Feng Z. Comparison of low-profiled visualized intraluminal support stent-assisted coiling and coiling only for acutely ruptured intracranial aneurysms: safety and efficacy based on a propensity score-matched cohort study. Neurosurgery. (2020) 87:584–91. 10.1093/neuros/nyaa110 32415845

[8] Liu Y Wang F Wang M Zhang G. Comparison of stent-assisted coil placement and coiling-only for the treatment of ruptured intracranial aneurysms. Med Sci Monit. (2017) 23:5697–704. 10.12659/MSM.905107 29190261 PMC5719724

[9] Zhang Z Li Z. Endovascular treatment of ruptured very small intracranial aneurysms: complications, recurrence rate, and clinical outcomes. Front Neurol. (2022) 12:7. 35058874 10.3389/fneur.2021.767649 PMC8764134

[10] Jin Y Guo X Quan T Chen Z Liu C Guan S. Safety and efficacy of endovascular treatment for tiny ruptured intracranial aneurysms with low-profile visualized intraluminal support stents. Interv Neuroradiol. (2022) 3:159101992210799. 10.1177/15910199221079967 35147055 PMC10152828

[11] Cho YD Sohn CH Kang HS Kim JE Cho WS Hwang G. Coil embolization of intracranial saccular aneurysms using the low-profile visualized intraluminal support (LVIS^TM^) device. Neuroradiology. (2014) 56:543–51. 10.1007/s00234-014-1363-x 24740581

[12] Xue G Zuo Q Zhang X Tang H Zhao R Li Q. Safety and efficacy of stent-assisted coiling for acutely ruptured wide-necked intracranial aneurysms: comparison of LVIS stents with laser-cut stents. Chin Neurosurg Jl. (2021) 7:19. 10.1186/s41016-021-00237-1 33653398 PMC7927374

[13] Ni H Zhao LB Liu S Jia ZY Cao YZ Shi HB. The safety and efficacy of endovascular treatment for very small ruptured anterior communicating artery aneurysms: a large single-center experience with 81 consecutive cases. World Neurosurg. (2021) 152:e576–82. 10.1016/j.wneu.2021.06.025 34133994

[14] Cai K Zhang Y Shen L Ni Y Ji Q. Comparison of stent-assisted coiling and balloon-assisted coiling in the treatment of ruptured wide-necked intracranial aneurysms in the acute period. World Neurosurg. (2016) 96:316–21. 10.1016/j.wneu.2016.09.029 27647035

[15] Hong Y Wang YJ Deng Z Wu Q Zhang JM. Stent-assisted coiling versus coiling in treatment of intracranial aneurysm: a systematic review and meta-analysis. PLoS ONE. (2014) 9:e82311. 10.1371/journal.pone.0082311 24454690 PMC3893071

[16] Malhotra A Wu X Forman HP Grossetta Nardini HK Matouk CC Gandhi D. Growth and rupture risk of small unruptured intracranial aneurysms: a systematic review. Ann Intern Med. (2017) 167:26. 10.7326/M17-0246 28586893

[17] Malhotra A Wu X Forman HP Matouk CC Gandhi D Sanelli P. Management of tiny unruptured intracranial aneurysms: a comparative effectiveness analysis. JAMA Neurol. (2018) 75:27–34. 10.1001/jamaneurol.2017.3232 29159405 PMC5833486

[18] Orrù E. Complications of endovascular treatment of cerebral aneurysms. Eu J Radiol. (2013) 6:11. 10.1016/j.ejrad.2012.12.011 23332977

[19] Zhao B Tan X Yang H Zheng K Li Z Xiong Y. AMPAS study group. Stent-assisted coiling versus coiling alone of poor-grade ruptured intracranial aneurysms: a multicenter study. J Neurointerv Surg. (2017) 9:165–8. 10.1136/neurintsurg-2016-012259 26951385

[20] Zhang X Zuo Q Tang H Xue G Yang P Zhao R. Stent assisted coiling versus non-stent assisted coiling for the management of ruptured intracranial aneurysms: a meta-analysis and systematic review. J NeuroIntervent Surg. (2019) 11:489–96. 10.1136/neurintsurg-2018-014388 30842307

[21] Darkwah Oppong M Buffen K Pierscianek D Herten A Ahmadipour Y Dammann P. Secondary hemorrhagic complications in aneurysmal subarachnoid hemorrhage: when the impact hits hard. J Neurosurg. (2019) 3:1–8. 10.3171/2018.9.JNS182105 30684947

[22] Roh H Kim J Bae H Chong K Kim JH Suh S-I Kwon T-H Yoon W. Comparison of stent-assisted and no-stent coil embolization for safety and effectiveness in the treatment of ruptured intracranial aneurysms. J Neurosurg. (2019) 45:1–7. 10.3171/2019.5.JNS19988 31470411

[23] Bodily KD Cloft HJ Lanzino G Fiorella DJ White PM Kallmes DF. Stent-assisted coiling in acutely ruptured intracranial aneurysms: a qualitative, systematic review of the literature. AJNR Am J Neuroradiol. (2011) 32:1232–6. 10.3174/ajnr.A2478 21546464 PMC7966042

[24] Nishido H Piotin M Bartolini B Pistocchi S Redjem H Blanc R. Analysis of complications and recurrences of aneurysm coiling with special emphasis on the stent-assisted technique. American Journal of Neuroradiology. (2014) 35:339–44. 10.3174/ajnr.A3658 23907240 PMC7965758

[25] Ge H Lv X Yang X He H Jin H Li Y. Stent vs. enterprise stent for the treatment of unruptured intracranial aneurysms. World Neurosurg. (2016) 91:365–70. 10.1016/j.wneu.2016.04.057 27113398

[26] Zhang L Chen X Dong L Liu P Jia L Zhang Y. Clinical and angiographic outcomes after stent-assisted coiling of cerebral aneurysms with laser-cut and braided stents: a comparative analysis of the literatures. Front Neurol. (2021) 12:666481. 10.3389/fneur.2021.666481 33995263 PMC8116799

[27] Mokin M Primiani CT Ren Z Piper K Fiorella DJ Rai AT. Stent-assisted coiling of cerebral aneurysms: multi-center analysis of radiographic and clinical outcomes in 659 patients. J NeuroIntervent Surg. (2020) 12:289–97. 10.1136/neurintsurg-2019-015182 31530655

[28] Li W Wang Y Zhang Y Wang K Zhang Y Tian Z. Efficacy of LVIS vs. enterprise stent for endovascular treatment of medium-sized intracranial aneurysms: a hemodynamic comparison study. Front Neurol. (2019) 10:522. 10.3389/fneur.2019.00522 31191428 PMC6546800

[29] Cagnazzo F Cappucci M Lefevre P-H Dargazanli C Gascou G Morganti R. Treatment of intracranial aneurysms with self-expandable braided stents: a systematic review and meta-analysis. AJNR Am J Neuroradiol. (2018) 39:2064–9. 10.3174/ajnr.A5804 30262643 PMC7655354

[30] Yang P Zhao K Zhou Y Zhao R Zhang L Zhao W. Stent-assisted coil placement for the treatment of 211 acutely ruptured wide-necked intracranial aneurysms: a single-center 11-year experience. Radiology. (2015) 276:545–52. 10.1148/radiol.2015140974 25822469

[31] Hong B Yang P Zhao R Huang Q Xu Y Yang Z. Endovascular treatment of ruptured tiny intracranial aneurysms. J Clin Neurosci. (2011) 18:655–60. 10.1016/j.jocn.2010.09.013 21414787

[32] Anokwute MC Braca JA Bohnstedt B DeNardo A Scott J Cohen-Gadol A. Endovascular treatment of ruptured tiny (63 mm) intracranial aneurysms in the setting of subarachnoid hemorrhage: a case series of 20 patients and literature review. J Clin Neurosci. (2017) 40:52–6. 10.1016/j.jocn.2017.01.011 28347681

[33] Wu P Ocak PE Wang D Ocak U Xu S Li Y. Endovascular treatment of ruptured tiny intracranial aneurysms with low-profile visualized intraluminal support device. J Stroke Cerebrovasc Dis. (2019) 28:330–7. 10.1016/j.jstrokecerebrovasdis.2018.09.052 30391328

[34] Fiorella D Arthur A Boulos A Diaz O Jabbour P Pride L. Final results of the US humanitarian device exemption study of the low-profile visualized intraluminal support (LVIS) device. J Neurointerv Surg. (2016) 8:894–7. 10.1136/neurintsurg-2015-011937 26391016

[35] Lim J Cho YD Hong N Lee J Yoo DH Kang HS. Follow-up outcomes of intracranial aneurysms treated using braided or laser-cut stents with closed-cell design: a propensity score-matched case-controlled comparison. J NeuroIntervent Surg. (2021) 13:434–7. 10.1136/neurintsurg-2020-016165 32817345

[36] King B Vaziri S Singla A Fargen KM Mocco J. Clinical and angiographic outcomes after stent-assisted coiling of cerebral aneurysms with enterprise and Neuroform stents: a comparative analysis of the literature. J NeuroIntervent Surg. (2015) 7:905–9. 10.1136/neurintsurg-2014-011457 25352581

[37] Yeon EK Cho YD Yoo DH Kim JE Kim KM Lee SH. Midterm outcomes after low-profile visualization endoluminal support or atlas stent-assisted coiling of intracranial aneurysms: a propensity score matching analysis. Neurosurgery. (2021) 89:862–6. 10.1093/neuros/nyab302 34382660

